# Intraurethral Catheter: Alternative Management for Urinary Retention in Patients With Benign Prostatic Hypertrophy

**DOI:** 10.1155/DTE.2.113

**Published:** 1995

**Authors:** Sulabha Punekar, Prem A. Ramkrishnan, Anand R. Kelkar, Jaydeep A. Date, Vasudeo R. Ridhorkar

**Affiliations:** 1 Department of Urology Seth G.S. Medical College and K.E.M. Hospital Bombay 400 012 India; 2 302, Manish Apartments Nehru Road Vile Parle (East) Bombay 400 057 India

## Abstract

A polyurethane intraurethral catheter (IUC) was used in 27 patients with benign prostatic hypertrophy who were unfit for surgery, or were awaiting surgery. All of them had previously had a periurethral catheter inserted. The IUC was inserted with a cystoscope under fluoroscopic control. Spontaneous voiding through the IUC resumed in 25 patients (93%) in the immediate postprocedure period. At the end of 6 months follow-up, the peak flow rates and the residual volumes estimated in 22 patients were satisfactory. Immediate complications included incontinence due to distal displacement in 2 patients and hematuria in one patient; long-term complications included mild encrustation of the IUC in 2 patients and calculus formation on the IUC in 1 patient. None of the patients had clinically significant urinary tract infection. The presence of the IUC did not compromise the subsequent transurethral resection of the prostate gland. We recommend the use of an IUC for up to 6 months in patients with urinary retention who are awaiting surgery or are unfit for surgery as an alternative to an indwelling urethral catheter.


*Diagnostic and Therapeutic Endoscopy*, 1995, Vol. 2, pp. 113–117
Reprints available directly from the publisher
Photocopying permitted by license only

© 1995 Harwood Academic Publishers GmbH
Printed in Singapore

### Intraurethral Catheter: Alternative Management for Urinary Retention in Patients with Benign Prostatic Hypertrophy

SULABHA PUNEKAR, PREM A. RAMKRISHNAN, ANAND R. KELKAR, JAYDEEP A. DATE and VASUDEO R. RIDHORKAR

*Department of Urology, Seth G.S. Medical College and K.E.M. Hospital, Bombay-400 012, India*

*(Received May 2, 1995; in final form, May 19, 1995)*

A polyurethane intraurethral catheter (IUC) was used in 27 patients with benign prostatic hypertrophy who were unfit for surgery, or were awaiting surgery. All of them had previously had a periurethral catheter inserted. The IUC was inserted with a cystoscope under fluoroscopic control. Spontaneous voiding through the IUC resumed in 25 patients (93%) in the immediate postprocedure period. At the end of 6 months follow-up, the peak flow rates and the residual volumes estimated in 22 patients were satisfactory. Immediate complications included incontinence due to distal displacement in 2 patients and hematuria in one patient; long-term complications included mild encrustation of the IUC in 2 patients and calculus formation on the IUC in 1 patient. None of the patients had clinically significant urinary tract infection. The presence of the IUC did not compromise the subsequent transurethral resection of the prostate gland. We recommend the use of an IUC for up to 6 months in patients with urinary retention who are awaiting surgery or are unfit for surgery as an alternative to an indwelling urethral catheter.

**KEY WORDS:** Prostate, stents, urethral catheter, urinary retention, prostatic hyperplasia, bladder neck obstruction, prostatic hypertrophy

### INTRODUCTION

Retention of urine is one of the common clinical presentations of benign prostatic hypertrophy (BPH). For decades this was managed with an indwelling urethral catheter. In many instances the indwelling urethral catheter has to be left in place for an extended period of time either because the patient is unfit for surgery, or because his condition requires that surgery be postponed. Also, in many centers, there is a waiting list of patients for prostatectomy, and the urethral catheter remains indwelling for weeks or even months.

An indwelling catheter is a common cause of urinary tract infections and urethral stricture and is associated with nearly a 3-fold increase in mortality among hospitalized patients (1,2). Men with indwelling catheters have low self-esteem, are unable to perform sexually, and have difficulty in discharging their day-to-day activities.

The polyurethane double Malecot intraurethral catheter (IUC) is a simple physiological prosthesis that obviates many of the difficulties faced with an indwelling intraurethral catheter (3). We present here our study of 27 patients in whom urinary retention caused by BPH was managed with an IUC.

Address for correspondence: Dr. S. V. Punekar, 302, Manish Apartments, Nehru Road, Vile Parle (East), Bombay 400 057, India.

### MATERIALS AND METHODS

Twenty-seven patients with retention of urine caused by BPH were offered the IUC as an alternative treatment instead of an indwelling urethral catheter. These patients were either at high risk for complications of anesthesia ($n$ = 22) or had a potentially long waiting period for prostatectomy ($n$ = 5). In a institution like ours serving a large segment of the population, the waiting period for transurethral resection of the prostate or prostatectomy often extends up to 6 to 8 months. Preprocedural assessement included subjective evaluation of bladder outflow obstruction, physical examination, cystourethroscopy, and subjective evaluation of sexual function.

A 16 F polyurethane double Malecot type of IUC manufactured by Angiomed was used. It is available in 35- to 70-mm lengths. It consists of a crown, the proximal basket, and a distal basket (Fig. 1). A nonabsorbable (nylon/silk) 0.20-mm suture is connected to the distal end of the IUC.

The procedure was carried out under local anesthesia (installation of Lignocaine jelly) on the uroradiology table. The length of the prostatic urethra was determined by preliminary cystourethroscopy. The IUC was placed in the distal end of a 23.5 cystoscope sheath and introduced into the bladder (Fig. 2). The IUC was expelled into the bladder with the use of the obturator. Under fluoroscopic control, the IUC was placed in the prostatic urethra with the distal end of the IUC positioned just beyond the verumontanum by pulling the nylon string attached to it (Fig. 2). The position was confirmed by cystoscopy and fluoroscopy. The nylon thread was kept long for 24 hours. Postprocedural micturition was closely observed immediately after treatment, and the patients were discharged from the hospital after 24 hours. All patients were given cotrimoxazole for 7 days. In the 2 initial patients we performed cystourethrography to confirm the position of the IUC (Fig. 3).

Follow-up visits were scheduled at 1, 3, and 6 months postoperatively. Postoperative assessement included eval-

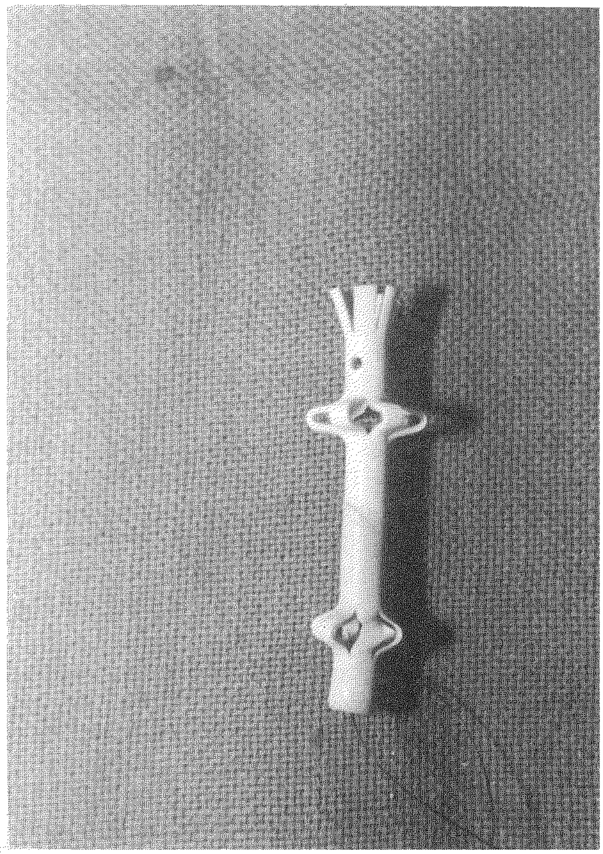

**Figure 1** The intraurethral catheter.

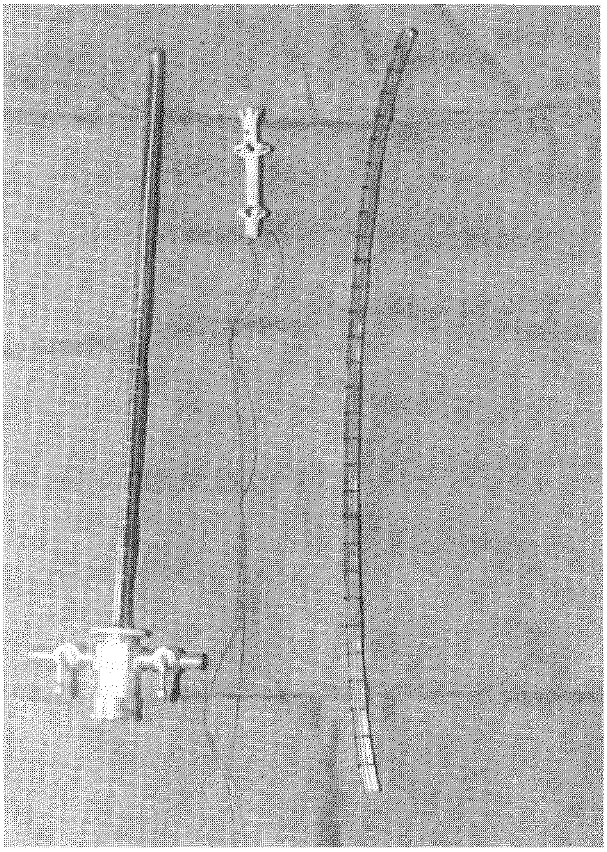

**Figure 2** The instrumentation set.

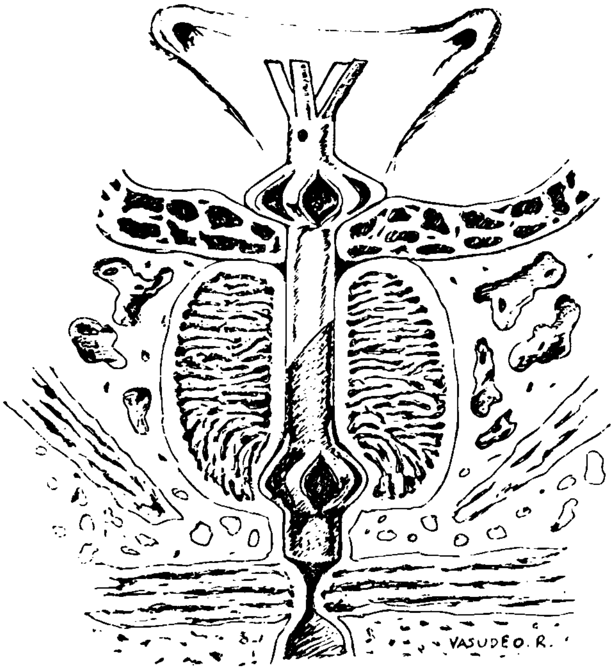

**Figure 3** The intraurethral catheter *in situ*.

uation of subjective symptoms, uroflowmetry, measurement of residual volume, and subjective evaluation of sexual function. Urine cultures were taken weekly during the first 2 weeks. At the end of 6 months 5 patients were lost to follow-up. Of the remaining 22 patients, 17 were subjected to transurethral resection of the prostate. Four patients refused further therapy; one died of cardiac illness.

### RESULTS

At the preoperative cystoscopic examination 22 patients (81%) had predominantly lateral lobe hyperplasia while 5 patients (19%) had median lobe hyperplasia. In addition, 1 patient had a previous undetected small vesical calculus, which was removed before the IUC was implanted. Before the episode of urinary retention 4 patients (15%) reported normal erection and antegrade ejaculation.

Spontaneous voiding was reestablished in 25 patients (93%) while the other 2 patients in whom the IUC had slipped into the bladder developed urinary retention. After repositioning of the IUC these 2 patients also had satisfactory voiding. The urinary flow parameters reported in Table 1 show satisfactory mean peak flow rates and mean residual volumes, which were maintained up to the last follow-up.

Six patients (22%) developed urinary frequency, which resolved on its own over a period of 2 to 3 weeks. None of the patients in whom the IUC was retained developed clinically significant urinary tract infections. During the follow-up period, 5 patients (19%) had an asymptomatic urinary tract infection with *Proteus*. Appropriate antibiotics were administered.

Four of our patients who were sexually active before development of retention of urine reported resumption of normal sexual activities after placement of the IUC. However, all of them reported retrograde ejaculation.

Immediate complications occurred in 3 patients (11%). These included incontinence secondary to the slipping of the IUC below the verumontanum in 2 patients and hematuria in another patient. Continence was regained after repositioning of the IUC, while hematuria resolved spontaneously within 24 hours. Long-term complications (i.e., at end of 6 months) occurred in 3 patients (11%). These included mild encrustation of the IUC in 2 patients and severe encrustation leading to calculus formation on the IUC in 1 patient (Fig. 4). The severely encrusted IUC had to be removed surgically through the suprapubic route, because the IUC had slipped into the bladder, and the patient had not come for follow-up for more than 8 months.

At the end of 6 months, 5 patients were lost to follow-up. Of the remaining 22 patients, 17 patients were subjected to transurethral resection of the prostate. At the time of transurethral resection there was mild cystitis in 3 patients and congestion of the prostate in 2 patients. The transurethral resections were unremarkable.

### DISCUSSION

The definitive surgery of many patients with retention of urine caused by BPH is often delayed for several weeks or even months because the patients may be suffering from concurrent severe medical illness that preclude anesthesia or there may be a long waiting period before surgery.

Insertion of a urethral catheter is the simplest form of urinary diversion, which is commonly used in such a clinical setting. However, prolonged catheterization is associated with a number of complications such as urosepsis and urethral stricture formation (4). Alteration in body image with associated depression and the discomfort related to the catheter are other factors that complicate indwelling urethral catheterization (5). Thus, an alternative modality for the management of urinary retention caused by an enlarged prostate, which permits a catheter-free existence, would be a valuable addition in the therapeutic armamentarium of the urologist.

In 1980, Fabian (6) pioneered the use of an intraurethral stent in the management of bladder outflow obstructions. This was a steel spiral placed in the prostatic urethra with an anchoring tail in the bulbar urethra. Subsequently Nissenkorn and Richter (3) reported the use of the double Malecot type IUC made of polyurethane, with excellent results. In 1992 we began to use this type of catheter. Attractive features of the IUC are its simple design, ease of insertion, positioning within the urethra under direct vision, and relatively inexpensive cost. The procedure can be performed under local anesthesia without supplemen-

**Table 1** Pre-and Postoperative Urinary Flow Parameters (Means ± SD)

| | | Follow-up | | |
|---|---|---|---|---|
| | *Preoperative* | *1 mo* | *3 mo* | *6 mo* |
| No. of patients assessed | 27 | 18 | 18 | 16 |
| Peak flow rate (ml/s) | | 15.1 + 4.2 | 13.6 + 3.4 | 13.2 + 3.6 |
| Voided volume (ml) | | 196 + 80 | 204 + 71 | 202 + 82 |
| Residual volume (ml) | | 43 + 64 | 40 + 58 | 33 + 62 |

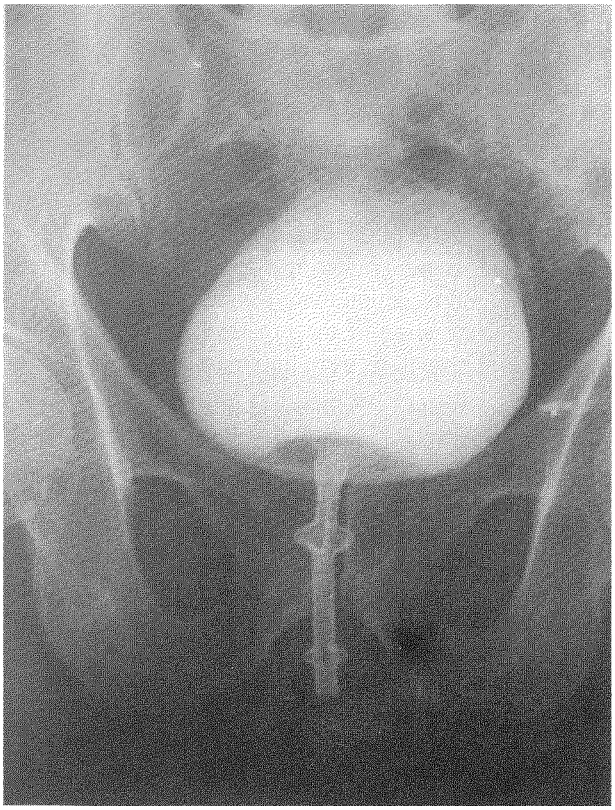

**Figure 4** Voiding cystourethrogram with the IUC in place.

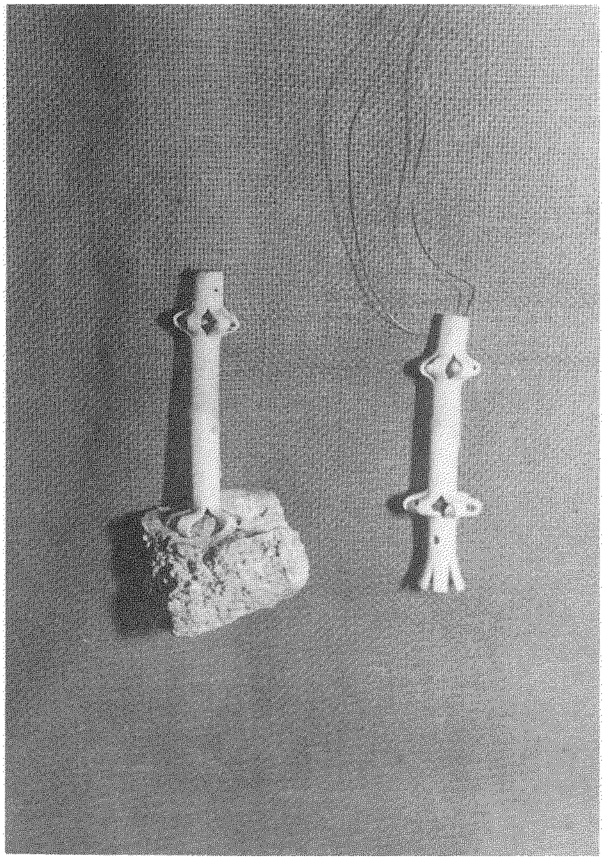

**Figure 5** Calculus formation on the IUC.

tal intravenous sedation and analgesia. The operative time is only 20 to 30 minutues.

The therapeutic benefits of an IUC are apparent. The majority of patients void satisfactorily without the need for external tubing or drainage. In our study 93% of the patients could void spontaneously after placement of an IUC. Satisfactory mean peak flow rates were obtained in all patients and the improvement seen from the first follow-up was maintained until the 6th month follow-up. Mean residual urine volume remained low at both the short- and long-term follow-ups.

Because patients are catheter-free, their day-to-day activities as well as social lives are not affected. This is particularly significant in the context of a conservative social milieu such as that in Indian, wherein a patient with an indwelling urethral catheter could be a social outcast.

The IUC does not hinder routine sexual activity in these patients. All of our patients who were sexually active before the insertion of the IUC continued to remain so even after its insertion, although in all of them retrograde ejaculation persisted. A major European multicenter study recently reported major positive effects on sexual function after prostatic stent placement (7). In their study, patients who reported antegrade ejaculation preoperatively continued to have antegrade ejaculation after stent placement.

After placement of the IUC, continence is maintained in the majority of the patients. The common causes of incontinence are slipping of the IUC distal to the verumontanum and urge incontinence secondary to urinary tract infection. Repositioning of the IUC under cystoscopic control and treatment of infection restores continence in these patients. The use of an appropriate sized IUC (length of IUC) as determined by cystourethroscopy reduced the incidence of distal migration and hence incontinence in our study.

It is important to conduct long-term follow-up of these patients with urine culture and plain frontal supine X-ray of the abdomen so that significant urinary tract infections and other complications such as major encrustation of the IUC can be detected and managed early.

Recently with newer technology developed in the biliary tract and blood vessels, there are reports advocating the use of permanent prostatic stents such as titanium stents and the UroLume stent (8–11). Although the success rates achieved are satisfactory, these stents are expensive and long-term results are not yet known.

### CONCLUSIONS

The intraurethral catheter is a simple device that alleviates urinary retention in patients with benign prostatic hypertrophy. The IUC can be placed under local anesthesia with ease, has minimal morbidity, and is relatively free of complications. The majority of the patients have a satisfactory voiding pattern and remain fully continent. By obviating the need for a periurethral catheter, the IUC allows the patient to discharge his day-to-day activities comfortably, as well as resume normal coital habits. Thus, the IUC is a viable alternative to an indwelling urethral catheter in patients with urinary retention caused by BPH, who are unfit for surgery due to medical illness or are awaiting definitive surgery.

### ACKNOWLEDGMENTS

We wish to thank Dr. P. M. PAI, The Dean, Seth G.S. Medical College and K.E.M. Hospital, Bombay, India, for permission and Smt. Hema Choughule for secretarial assistance.

### REFERENCES

1. Harley RW, Hooton RM, Culver DH. Nosocomial infection in U.S. hospitals 75–76. Estimated frequency by selected characteristics of patients. Am J Med 1981;70:947–951.
2. Muncie HL, Warren JW. Reasons for replacement of longterm urethral catheters: implications for randomized trials. J Urol 1990;143:507–510.
3. Nissenkorn I, Richter S. A new self-retaining intraurethral device: an alternative to an indwelling catheter in patients with uri nary retention due to infravesical obstruction. Br J Urol 1990; 65:197–201.
4. Ouslander JG, Greengold B, Chen S. Complications of chronic indwelling urinary catheters among male nursing home patients: a prospective study. J Urol 1987;138:1191–1193.
5. Morgantaler A, DeWolf WC. A self expanding prostatic stent for bladder outlet obstruction in high risk patients. J Urol 1993;150:1636–1640.
6. Fabian KM. Der intraprostatische "Partielle Katheter" (Urologische Spirale). Urologe A 1980;19:236–239.
7. Giorgio G, Pansadoro V, Montorsi F, et al. A modified prostatic UroLume Wallstent for healthy patients with symptomatic benign prostatic hyperplasia: a European multicenter study. Urology 1994;44:364–370.
8. Kaplan SA, Merrill DC, Mosely WG, et al. Titanium intraprostatic stent: United States experience. J Urol 1993;150:1624–1629.
9. Milroy E, Chapple CR. UroLume stent in management of benign prostatic hyperplasia. J Urol 1993;150:1630–1635.
10. Nordling J, Oveson H, Poulsen AL. The intraprostatic spiral: clinical results in 150 consecutive patients. J Urol 1992; 147: 645–650.
11. Guazzoni G, Bergamaschi I, Montorsi I, et al. Prostatic UroLume Wallstent for BPH patients at poor operative risk: clinical, uroflowmetric and ultrasonographic patterns. J Urol 1993; 150:1641–1647.

